# Molecular Identification and Characterization of Hydroxycinnamoyl Transferase in Tea Plants (*Camellia sinensis* L.)

**DOI:** 10.3390/ijms19123938

**Published:** 2018-12-07

**Authors:** Chi-Hui Sun, Chin-Ying Yang, Jason T. C. Tzen

**Affiliations:** 1 Graduate Institute of Biotechnology, National Chung Hsing University, Taichung 40227, Taiwan; bettychi2121@gmail.com; 2 Department of Agronomy, National Chung Hsing University, Taichung 40227, Taiwan

**Keywords:** tea, hydroxycinnamoyl transferase, abiotic stress, ABA signaling, hormone

## Abstract

Tea (*Camellia sinensis* L.) contains abundant secondary metabolites, which are regulated by numerous enzymes. Hydroxycinnamoyl transferase (HCT) is involved in the biosynthesis pathways of polyphenols and flavonoids, and it can catalyze the transfer of hydroxyconnamoyl coenzyme A to substrates such as quinate, flavanol glycoside, or anthocyanins, thus resulting in the production of chlorogenic acid or acylated flavonol glycoside. In this study, the *CsHCT* gene was cloned from the Chin-Shin Oolong tea plant, and its protein functions and characteristics were analyzed. The full-length cDNA of CsHCT contains 1311 base pairs and encodes 436 amino acid sequences. Amino acid sequence was highly conserved with other HCTs from *Arabidopsis thaliana*, *Populus trichocarpa*, *Hibiscus cannabinus*, and *Coffea canephora*. Quantitative real-time polymerase chain reaction analysis showed that *CsHCT* is highly expressed in the stem tissues of both tea plants and seedlings. The *CsHCT* expression level was relatively high at high altitudes. The abiotic stress experiment suggested that low temperature, drought, and high salinity induced *CsHCT* transcription. Furthermore, the results of hormone treatments indicated that abscisic acid (ABA) induced a considerable increase in the *CsHCT* expression level. This may be attributed to CsHCT involvement in abiotic stress and ABA signaling pathways.

## 1. Introduction

After water, tea has been the most widely consumed beverage worldwide for several thousand years because of its unique aroma and taste. Tea plants (*Camellia sinensis* L.) contain abundant specialized secondary metabolites such as polyphenolic compounds, found in the largest proportions in tea plants alkaloids, terpenoids, and amino acids [1]. Tea polyphenols account for 30% of the dry weight of tea leaves. They can be roughly divided into the following five categories: flavanols, flavonols, flavones, proanthocyanidins, and phenolic acids [2,3].

Hydroxycinnamoyl transferase (HCT) catalyzes the transfer of hydroxycinnamoyl moiety to receptor substrates such as shikimic acid, quinic acid, anthocyanins, flavanol glycoside, polyamine, and long-chain fatty acids. Plants under environmental stresses can induce the related gene expression involved in the phenylpropanoid metabolic pathway to generate various secondary metabolites that resist or adapt to environmental stresses [4]. The HCT involved in the phenylpropanoid pathway catalyzes shikimic acid and quinic acid to participate in the upstream pathway of lignin biosynthesis. Lignins in plant cell walls provide a physical defense that protects polysaccharides in cell walls from degradation by microorganisms [5]. Low temperature, high salinity, drought, mechanical injury, abscisic acids (ABAs), salicylic acid (SA), and hydrogen peroxide can induce *HcHCT* expression in *Hibiscus cannabinus*. HcHCT increases abiotic stress tolerance in plants [6]. In *Cucumis sativus*, *HCT* expression was reduced with pectinase treatment. In addition, directing the phenylpropanoid pathway to generate H-lignin caused p-coumaraldehyde accumulation [7].

HCT not only participates in secondary metabolite acylation but also regulates hypersensitive responses (HRs) in plants. HCT1806 or HCT4918 in *Zea mays* interacts with Rp1-D21 translated from resistance genes, thereby inhibiting HR generation. When pathogens attack plants, effectors secreted by the pathogens can change the protein structure of HCT1806 or HCT4918, which influences how they interact with Rp1-D21 and causes plants to generate HR. This prevents the spread of pathogens in local cell necrosis [8]. HCT is specific to a wide range of substrates such as gentisate, 3-hydroxybenzoate, hydroxyanthranilate, and protocatechuate, and competes with shikimic acid or quinate acid for the binding site on the enzyme, which in turn produces other acylation products [9,10,11].

In this study, to clarify the molecular characteristics of HCT in tea plants, we analyzed highly conserved domains in the amino acid sequences of HCT in *Arabidopsis thaliana*, *Nicotiana tabacum*, *H. cannabinus*, *Theobroma cacao*, and *Fragaria vesca*, designed degenerate primers for use in polymerase chain reaction (PCR), and cloned the genetic sequence of *CsHCT* from a Chin-Shin Oolong tea plant. Quantitative real-time PCR (qRT-PCR) was used to analyze *CsHCT* expression levels in the tissues of tea plants and seedlings. The results demonstrated a high level of *CsHCT* expression in the stem tissues of tea plants and seedlings. The amount of *CsHCT* transcribed in tea plants at various altitudes and in different seasons was also measured, and the results indicated that CsHCT expression levels were relatively high at high altitudes and at low temperatures. Moreover, an abiotic stress experiment revealed that low-temperature, drought, and high-salinity stresses induced *CsHCT* transcription. In addition, *CsHCT* expression increased with ABA treatment. Thus, this study concluded that CsHCT may be involved response to abiotic stress and ABA signaling pathways in tea plants.

## 2. Methods

### 2.1. Plant Materials and Growth Conditions

This study used the tea [*C. sinensis* (L.) Kuntze] cultivar Chin-Shin Oolong in these experiments. These were obtained from a tea seed germination farm operated in Nantou in central Taiwan. The seedlings were 1–2 years old with a height of 40–50 cm. The tea plant (>10 years old) samples were obtained from a tea farmer in Nantou County. The samples were collected between 2015 and 2016 and consisted of buds and young leaf (YL), old leaf (OL), young stem (YS), and old stem (OS) tissues. They were obtained from high-mountain tea plantations in the Alishan area of Chiayi County. The tea plantations were located at altitudes between 700 and 1300 m, and all the tea plants were >10 years old.

### 2.2. Bioinformatics Analysis of the CsHCT Gene and Amino Acid Sequence

This study analyzed the highly conserved domains of the HCT protein sequence in *A. thaliana*, *N. tabacum*, *H. cannabinus*, *T. cacao*, and *F. vesca*, designed a degenerate primer, and used the cDNA of the Chin-Shin Oolong tea plant as the template to perform PCR for obtaining the gene fragment sequence of *CsHCT*. The SMARTerTM RACE cDNA amplification kit (Clontech Laboratories, Inc., Mountain View, CA, USA) was used to expand 5′-end and 3′-end cDNA sequences. The full-length cDNA sequence of *CsHCT* was obtained after sequencing.

In the bioinformatics analysis conducted on the amino acid sequence of CsHCT, the ExPASy Translate tool (https://web.expasy.org/translate/) (access on July, 2016) was used for estimating the amino acid sequence translated by a nucleotide. The ExPASy Compute pI/Mw tool (https://web.expasy.org/compute_pi/) (access on July, 2016) was used to estimate the protein molecular weight and isoelectric point. Multiple sequence alignments were performed using the EMMA function of the EMBOSS explorer (http://www.bioinformatics.nl/emboss-explorer/) (access on July, 2016) and the BLOSUM50 scoring matrix, and GeneDoc software was used to compare the results. Subsequently, Motif Scan (https://myhits.isb-sib.ch/cgi-bin/motif_scan) (access on July, 2016) was used to predict the structural and functional protein regions. This study used the National Center for Biotechnology Information (https://www.ncbi.nlm.nih.gov/) (access on July, 2016) and Phytozome v10.3 (https://phytozome.jgi.doe.gov/pz/portal.html) (access on July, 2016) websites to obtain the protein sequences of clade Vb of BAHD (BEAT, benzylalcohol-O-acetyltransferase; AHCT, anthocyanin O-hydroxycinnamoyltransferase; HCBT, anthranilate N-hydroxycinnamoyl-benzoyltransferase; and DAT, deacetylvindoline 4-O-acetyltransferase) acyltransferase from *A. thaliana*, *Oryza sativa*, *Populus trichocarpa*, *Coffea canephora*, and *H. cannabinus*. Sequence alignment was performed using the ClustalW model. A phylogenetic tree was constructed using the MEGA6 software, after which statistical analysis was conducted through the neighbor joining method. The 1000 iterations of the tree algorithm were performed using the bootstrap method. SignalP (http://www.cbs.dtu.dk/services/SignalP/) (access on July, 2016) was used to predict whether a protein signal peptide existed. The Hphob./Kyte and Doolittle method of the ExPASy ProtScale (https://web.expasy.org/protscale/) (access on July, 2016) was adopted for predicting whether the proteins were hydrophilic or hydrophobic. The subcellular localization of proteins was predicted using WoLF PSORT (https://www.genscript.com/wolf-psort.html) (access on July, 2016). 

### 2.3. Abiotic Stress and Hormone Treatments on Tea Seedlings

The 1-year-old tea seedlings were treated with low temperature, high temperature, high salinity, and drought. Treatment conditions were as follows. The low- and high-temperature stresses were 5 °C and 35 °C, respectively. The seedlings were watered on optimum level and treated with the stresses for 12 h. Under the high-salinity stress, the seedlings were given 50 mL of 300 mM NaCl at 20 °C per day, whereas under the drought stress, they were not given additional water. The two treatments lasted for 5 days. In the control group, the seedlings were on optimum level watered at 20 °C for 5 days. After the treatments, samples were collected and preserved in a −80 °C environment for subsequent analysis. For hormone treatments, 100 µM solutions of ABA, SA, methyl jasmonate (MeJA), and 1-aminocyclopropane-1-carboxylic acid (ACC) solutions were prepared and sprayed on the YLs of the seedlings. After waiting for 6 h, the samples were collected and preserved in a −80 °C environment for subsequent analysis.

### 2.4. Extraction of Total RNA and qRT-PCR

In the experiment, 0.2 g of tea leaf samples was ground into powder in liquid nitrogen, and the total RNA was extracted using the Plant Total RNA Purification Kit (GeneMark, Taichung, Taiwan). The Moloney Murine Leukemia Virus (MMLV) first-strand synthesis kit (Gene DireX, Las Vegas, NV, USA) was used for reaction of 2 µg of total RNA. In the reaction solution, 1 µL of Oligo dT (1 µg/µL) was mixed with 1 µL of 10 mM dNTP to react for 10 min at 70 °C and for 5 min at 4 °C. After the reaction, 4 µL of 5× reaction buffer, 2 µL of 0.1 M Dithiothreitol (DTT), 1 µL of diethylpyrocarbonate (DEPC) H_2_O, and 1 µL of MMLV reverse transcriptase were sequentially added into the solution for 1 h of reaction at 37 °C and 10 min of reaction at 65 °C, after which the reaction was terminated. Subsequently, 80 µL of DEPC H_2_O was added into a 0.2-mL microcentrifuge tube to perform qRT-PCR on the obtained cDNA.

For qRT-PCR, the cDNA was amplified using the CFX ConnectTM Real-Time System (Bio-Rad, Hercules, CA, USA), and data were analyzed using Bio-Rad CFX Manager 3.1. The *18S rRNA* of the tea leaf samples were used as the internal control to normalize cDNA levels. The reaction conditions were as follows: 5 min at 94 °C; 15 s of 45 circulations at 94 °C, 60 °C, and 72 °C each; and finally, 10 min at 72 °C. Nonspecific products or primer dimers were identified based on their lower melting temperature than that of the specific amplicon. The primers used for qRT-PCR analyses were as follows: *CsHCT* forward sequence 5′-caaattaaccaaggaccaactcaac-3′ and reverse sequence 5′-tgtaattgaccatgttcccatcttc-3′; and *18S rRNA* forward sequence 5′-ccgctggcaccttatgagaa-3′ and reverse sequence 5′-tttcagccttgcgaccatact-3′. The qRT-PCR experiments were repeated at least 3 times each biologically independently, and the data shown are average values. Statistical analyses were performed using Statistical Analysis System (SAS) 9.4 software.

## 3. Results

### 3.1. Bioinformatics Analysis of the CsHCT Gene and Amino Acid Sequences of C. sinensis L.

*C. sinensis* L. contains numerous polyphenolic compounds that provide multiple health benefits. To understand the role of HCT in the reaction of acylated flavonol glycosides, the *CsHCT* gene was cloned from the Chin-Shin Oolong tea plant. The *CsHCT* gene has cDNA of length 1552 bp that includes 35-bp and 182-bp 5′ and 3′ untranslated regions (excluding a poly-A tail). The open reading frame includes 1311 nucleotide sequences, which can encode 436 amino acid sequences (GenBank accession number: MH271107).

**Figure 1:**
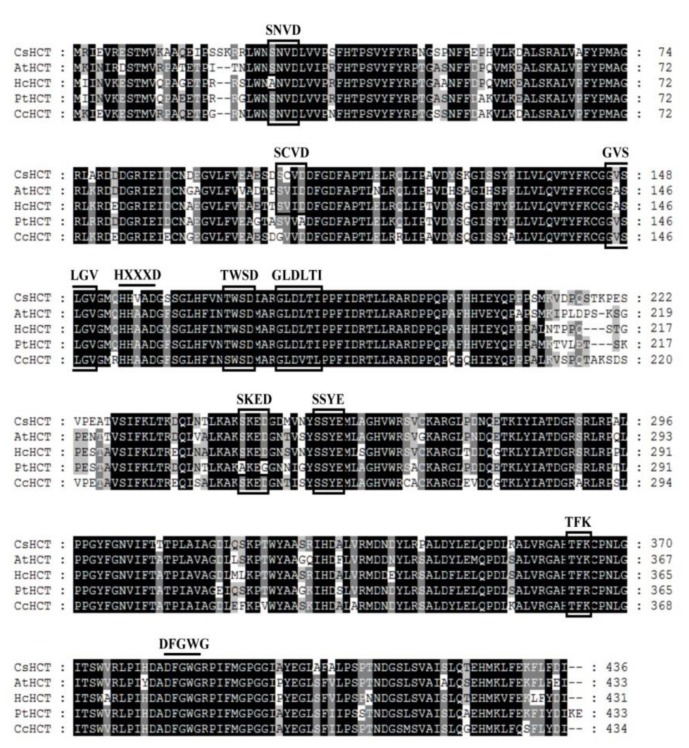
Alignment of deduced amino acid sequences of CsHCT with other putative HCTs. Black and gray shadings indicate conservation of 100% and at least 80%, respectively. Amino acid residues enclosed by squares correspond to consensus sequences of SXXD, SXXE, HXXXD, GVXXGV, TXXD, GLXXTI, DFGWG, etc. AtHCT, hydroxycinnamoyl-CoA shikimate/quinate hydroxycinnamoyl transferase was from *A. thaliana* (NP_199704); HcHcT, putative hydroxycinnamoyl-CoA shikimate/quinate hydroxycinnamoyl transferase was from *H. cannabinus* (AFN85668); PtHCT, quinate O-hydroxycinnamoyl transferase/shikimate O-hydroxycinnamoyl transferase was from *P. trichocarpa* (ACC63882); and, CcHCT, hydroxycinnamoyl-CoA shikimate/quinate hydroxycinnamoyl transferase was from *C. canephora* (ABO47805). Alignment was performed using the ClustalW algorithm. Sequence identities with CsHCT were as follows: AtHCT, 79.5%; HcHCT, 81.5%; PtHCT, 81.9%; and, CcHCT, 82.1%.

Sequence alignment analysis was performed on the amino acid sequence of CsHCT of *C. sinensis* L. and the HCT sequences of *A. thaliana* (AtHCT), *H. cannabinus* (HcHCT), *P. trichocarpa* (PtHCT), and *C. canephora* (CcHCT). Using global alignment and the BLOSUM50 scoring matrix, the similarities between CsHCT and AtHCT, HcHCT, PtHCT, and CcHCT were found to be 79.5%, 81.5%, 81.9%, and 82.1%, respectively. CsHCT and the HCT of other higher plants exhibited the sequences HXXXD and DFGWG, which are the conserved sequences of BAHD acyltransferase (Figure 1). Motif scanning was then performed to predict the amino acid sequence of CsHCT, and the results indicated that the N-terminus of its protein possesses the predicted N-myristoylation, casein kinase II phosphorylation, and protein kinase C phosphorylation sites.

**Figure 2:**
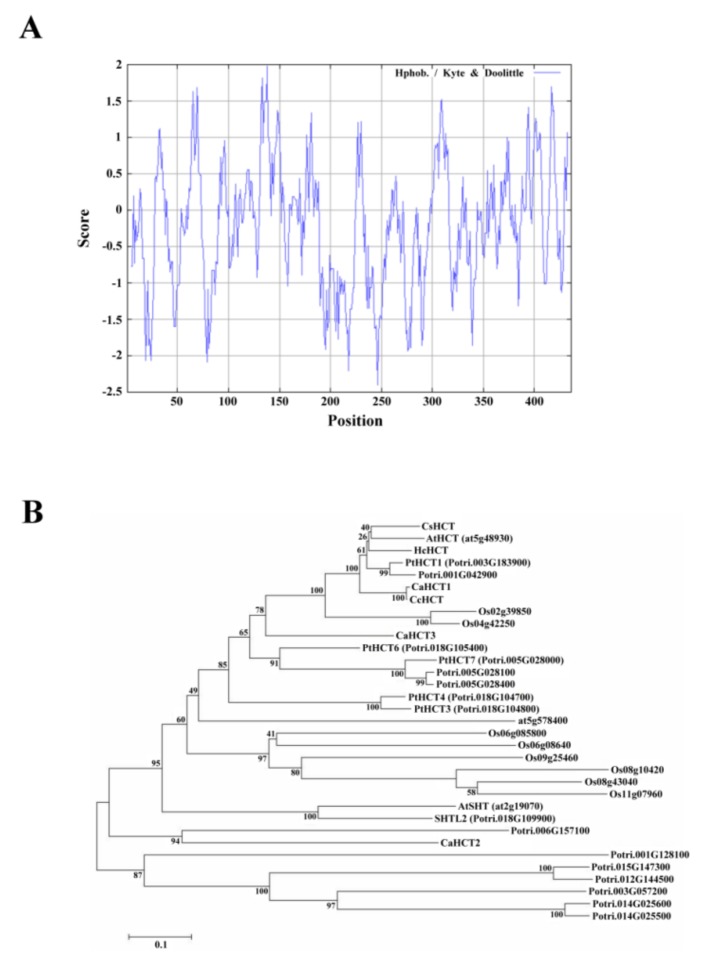
Hydropathy plot and phylogenetic tree analysis for CsHCT. (**A**) Hydropathy plot of CsHCT using the Kyte–Doolittle method with a window size of 436 amino acids. The window position values indicated on the x-axis of the graph reveal the average hydropathy of the entire window, with the corresponding amino acids as the middle element of that window. Plots above 0 (zero) in the graph indicate hydrophobic regions in the protein, and those below 0 (zero) indicate hydrophilic regions. (**B**) Unrooted phylogram of members of the HCT protein family. Phylogenetic tree of AtSHT (at2g19070), AtHCT (at5g48930), and HXXXD-type acyl transferase family protein (at5g57840) proteins in *Arabidopsis*; transferase family protein (Os02g39850; Os04g42250; Os06g08580; Os06g08640; Os08g10420; Os08g43040; Os09g25460; and Os11g07960) in *O. sativa*; PtHCT1 (Potri.003G183900), PtHCT3 (Potri.018G104800), PtHCT4 (Potri.018G104700), PtHCT6 (Potri.018G105400), PtHCT7 (Potri.005G028000), SHTL2 (Potri.018G109900), Shikimate O-hydroxycinnamoyl transferase (Potri.005G028100 and Potri.005G028400), transferase family protein (Potri.006G157100; Potri.015G147300; Potri.003G057200; Potri.001G042900; Potri.001G128100; Potri.014G025500; and Potri.012G144500), anthranilate N-hydroxycinamoyl/benzoyltransferase-like protein (Potri.014G025600) in *P. trichocarpa*; CaHCT1 (CAJ40778), CaHCT2 (CAT00082), CaHCT3 (CAT00081), CcHCT (ABO77955) in *C. canephora*; and HcHCT (AFN85668) in *H. cannabinus*. The phylogenetic tree was constructed by the Neighbor Joining algorithm implemented in the MEGA 6 software package. The 1000 iterations of the tree algorithm were performed using the bootstrap method.

Compute pI/Mw was used to analyze the amino acid sequence of CsHCT; its molecular weight and isoelectric point were predicted to be 48.53 kDa and 5.86, respectively. Analysis using SignalP failed to identify a signal peptide in CsHCT. The hydrophilicity and hydrophobicity of the protein were then analyzed using ProtScale, and the results demonstrated that the amino acid sequence did not possess an apparent hydrophobic end. The distribution of the hydropathy indices indicated that CsHCT is a hydrophilic protein (Figure 2A). Tuominen et al. (2011) reported that HCT is a member of clade Vb of the BAHD acyltransferase family. To understand the phylogenetic relationships between CsHCT and other clade Vb members, the MEGA6 software was used to perform neighbor joining. This generated a phylogenetic tree for the CsHCT of *C. sinensis* L. and the proteins of clade Vb members in *A. thaliana*, *O. sativa*, *P. trichocarpa*, *C. canephora*, and *H. cannabinus*. The results demonstrated that CsHCT and AtHCT had the closest phylogeny (Figure 2B); thus, they may have similar biochemical characteristics.

### 3.2. High Level of CsHCT Expression in the Stem Tissues of Tea Plants and Seedlings

**Figure 3:**
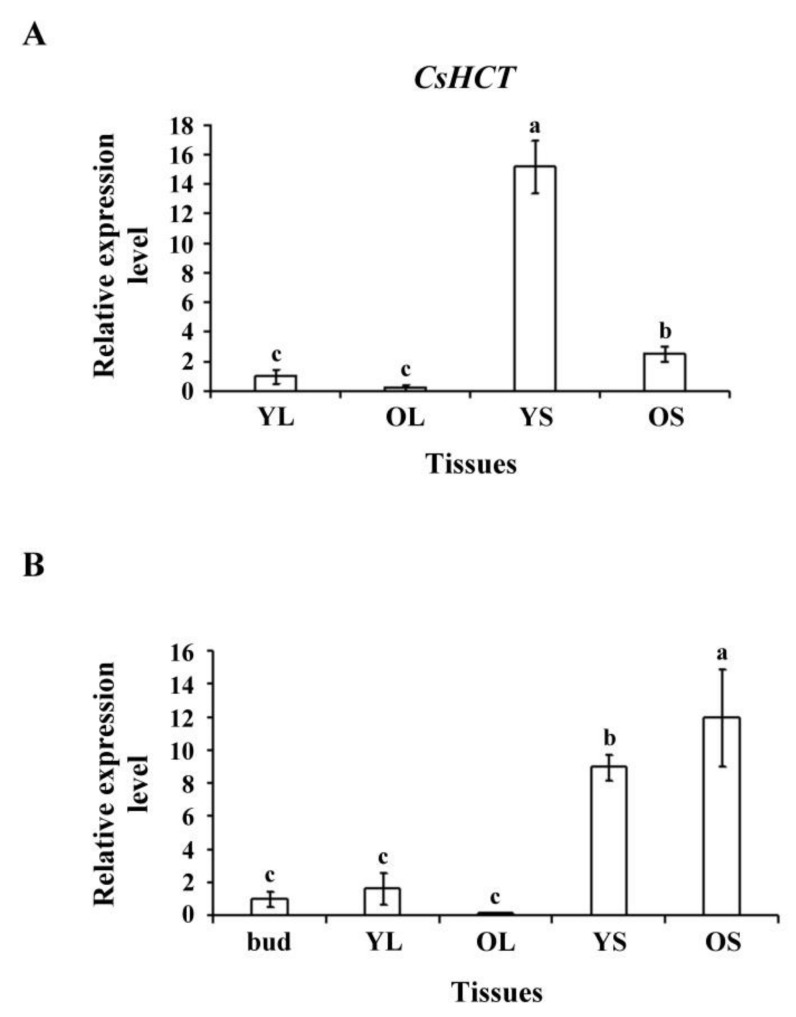
CsHCT transcription levels in various tissues of tea plants; qRT-PCR analysis of CsHCT transcription levels in various tissues of (**A**) tea seedlings and (**B**) tea plants. Total RNA was isolated from young leaf (YL), old leaf (OL), young stem (YS), old stem (OS), and buds. CsHCT transcription levels were determined. Relative amounts of transcripts were calculated and normalized to that of *18S rRNA*. Values represent means ± SD from three biologically independent experiments. Values with different letters are significantly different at *p* < 0.05, according to a post hoc least significant difference (LSD) test.

To assess *CsHCT* expression in tissues of tea plants and seedlings, we selected YL, OL, YS, and OS tissues from 1-year-old tea seedlings and extracted the total RNA. Reverse transcriptase was then used to synthesize the first cDNA for qRT-PCR analysis. The results indicated that the *CsHCT* expression level was the highest in YS tissues, followed by OS tissues, buds, and leaves in tea seedlings (Figure 3A). The transcription level was the highest in OS tissues, followed by YS, YL, and OL tissues in tea plants (Figure 3B). Nonlignified YS tissues in tea seedlings exhibited the highest CsHCT expression level, but in tea plants, the CsHCT expression level was high in both OS and YS tissues. Moreover, CsHCT expression was evident in the buds and YL tissues of both tea plants and seedlings; however, expression levels remained lower than those in stem tissues (Figure 3).

### 3.3. CsHCT Transcription Levels in Oolong Tea Plants during Four Growing Seasons and at Different Altitudes

**Figure 4:**
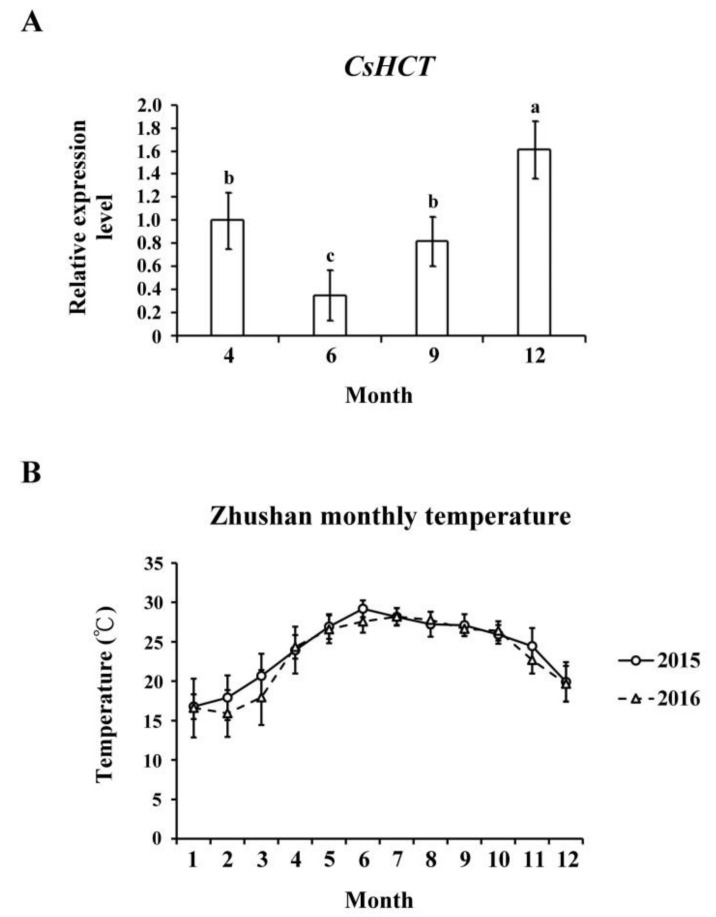
*CsHCT* transcription levels in four growing seasons. (**A**) The qRT-PCR analysis of CsHCT transcription levels in four growing seasons in the field. (**B**) Monthly average temperature in the Zhushan area. Total RNA was isolated from tissues of handpicked one-tip-two-leaf oolong tea samples collected in April, June, September, and December. Transcript levels of *CsHCT* were calculated and normalized to that of *18S rRNA*. Values represent means ± SD from five biologically independent experiments. Values with different letters are significantly different at *p* < 0.05, according to a post hoc least significant difference (LSD) test.

Temperature affects gene expression related to the secondary metabolism of plants, which regulates the generation of secondary metabolites and enables plants to adapt to environmental changes. To evaluate the influence of temperature on *CsHCT* expression, we used qRT-PCR to analyze its transcription levels in one-tip-two-leaf tissues of oolong tea plants during four seasons and compared their *CsHCT* expression. Specifically, the tea samples were collected from Zhushan, Nantou County, Taiwan in April, June, September, and December. The results showed that the *CsHCT* expression level of tea samples from December was the highest, and that of the tea samples from June was the lowest (Figure 4A). According to the average monthly temperature in Zhushan between 2015 and 2016 released by the Central Weather Bureau, *CsHCT* expression levels were negatively correlated with temperature (Figure 4B). *CsHCT* expression levels were high in low-temperature seasons.

**Figure 5:**
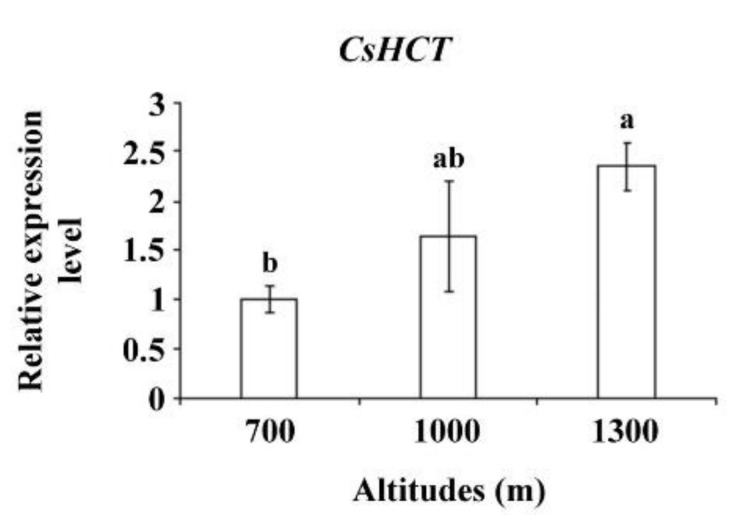
*CsHCT* expression levels at various altitudes; qRT-PCR analysis of *CsHCT* transcription levels in the Alishan area of Nantou County. Total RNA was isolated from the tissues of handpicked one-tip-two-leaf oolong tea samples collected at altitudes of 700, 1000, and 1300 m. Transcription levels of *CsHCT* were calculated and normalized to that of *18S rRNA*. Values represent means ± SD from three biologically independent experiments. Values with different letters are significantly different at *p* < 0.05, according to a post hoc LSD test.

Altitude influences temperature, humidity, and sunlight intensity. Temperature decreases as altitude increases. To further verify *CsHCT* expression, the tea samples collected from the Alishan area of Nantou County from altitudes of 700, 1000, and 1300 m and bud tissues were analyzed using qRT-PCR to determine CsHCT expression. The results demonstrated that the *CsHCT* expression level was the highest at 1300 m and decreased as the altitude decreased (Figure 5).

### 3.4. Effects of Abiotic Stresses and Hormone Signaling on CsHCT Transcript Levels in Oolong Tea Seedlings

**Figure 6:**
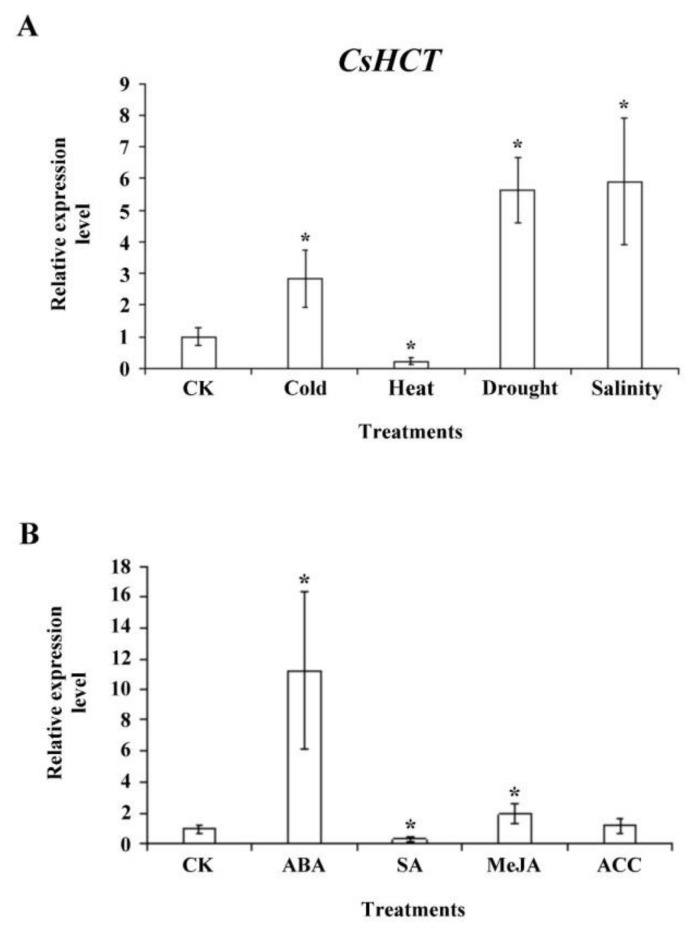
*CsHCT* expression levels under various abiotic stress and phytohormone treatment; qRT-PCR analysis of *CsHCT* transcription levels in tea seedlings after (**A**) abiotic stress and (**B**) phytohormone treatment. Total RNA was isolated from YLs of tea seedlings after cold treatment (5 °C) for 12 h, heat treatment (35 °C) for 12 h, drought (no water) for 3 days, and salt treatment (300 mM NaCl) for 5 days. The phytohormone treatment included treatment with 100 µM ABA, SA, MeJA, and ACC for 6 h. *CsHCT* transcription levels were calculated and normalized to that of *18S rRNA*. Values represent means ± SD from four biologically independent experiments. * *p* < 0.05, versus value in control check (CK) treatment (Student’s *t* test).

To investigate whether *CsHCT* expression is induced by abiotic stress, we exposed 1-year-old tea seedlings to stresses such as high temperature (35 °C), low temperature (5 °C), high salinity (300 mM NaCl), and drought; subsequently extracted the total RNA from one-tip-two-leaf tissues of oolong tea seedlings; and then performed qRT-PCR analysis. The results demonstrated that compared with the control group, *CsHCT* expression was higher at low-temperature stress and lower under high-temperature stress, thus verifying the association between *CsHCT* expression and temperature. Furthermore, *CsHCT* transcription levels also increased in response to the high-salinity and drought treatments (Figure 6A).

When a plant is under biotic or abiotic stresses, stress-related hormone signaling initiates the plant’s defense mechanisms and increases its stress tolerance. To assess whether CsHCT expression is induced by various stress hormone signals, we treated the 1-year-old tea seedlings with ABA, SA, MeJA, and the ethylene precursor ACC for 6 h and subsequently collected the one-tip-two-leaf tissues for analysis of *CsHCT* expression through qRT-PCR. The results indicated that *CsHCT* expression was induced under ABA and MeJA treatment and that the expression level was the highest in the ABA group. By contrast, the *CsHCT* expression level in the SA group was significantly lower than that in the control (Figure 6B). Accordingly, this study determined that CsHCT expression can be induced by abiotic stresses such as low temperature, high salinity, and drought, and inferred that CsHCT may be involved in the ABA signaling pathways.

## 4. Discussion

Acyltransferase in higher plants can catalyze transfer of acyl group to donor substrate. Acyl esters are produced as a result of the acyl group transfer from the donor substrate to the acceptor substrate [12]. Acyltransferase can be divided into two protein families, namely BAHD acyltransferase and serine carboxypeptidase-like acyltransferase, according to different donor substrates. HCT is categorized into the clade Vb of BAHD acyltransferase and characterized by its ability to catalyze various substrates.

Amino acid analysis results demonstrated that the structure and function of the CsHCT and HCT sequences of other higher plants were highly conserved (Figure 1). The amino acid domain was highly conserved in clade Vb that possessed a particularly conserved sequence SXXDL in the BAHD acyltransferase family [13]. However, whether the amino acid domain affects the catalytic function of enzymes remains to be investigated and verified. BAHD acyltransferase acts mainly in the cytoplasm of plant cells. Its substrate, acyl coenzyme A thioesters, can perform biosynthesis in various cell organs and be transferred to the cytoplasm by the transporter on the cell membrane, which facilitates the catalytic reaction of the BAHD acyltransferase [14]. After analyzing the cellular localization of CsHCT, we inferred that CsHCT is primarily located in the cytoplasm, and the amino acid sequence does not possess a signal peptide or apparent hydrophobic end (Figure 2A). Therefore, this study inferred that CsHCT primarily reacts in the cytoplasm for catalytic reaction.

Our data determined that the *CsHCT* expression level in the YS tissues of tea seedlings was higher than that in the OS and bud tissues, whereas the *CsHCT* expression level in tea plants was higher in OS and YS tissues (Figure 3). Studies on *Trifolium pretense* have shown that *HCT1* is primarily expressed in stem and flower tissues, whereas *HCT2* is mainly expressed in leaf and flower tissues, which indicates that HCT1 and HCT2 have different catalytic functions in diverse plant tissues [15]. *P. trichocarpa* possesses seven *PtrHCTs* that can be expressed in the tissues of various plant parts and exhibit differences with respect to their relative performance. In particular, *PtrHCT1* and *PtrHCT6* are primarily expressed in stem tissues, whereas *PtrHCT3* has a higher level of expression in leaf tissues [16]. In this study, nonlignified YS tissues of tea seedlings were found to contain a relatively large amount of *CsHCT* transcripts. The *CsHCT* expression level in YS tissues was eight times higher than that in OS tissues. This indicated that secondary metabolites and the expression of related biosynthesis genes in tissues vary according to the growth stages of *C. sinensis* L.

When plants are under environmental stresses, the expression of genes related to the biosynthesis of secondary metabolites is induced, which results in the generation and accumulation of compounds such as phenylpropanoid, flavonoids, and anthocyanins that can increase plants’ tolerance to stresses [4]. Our results demonstrated that the *CsHCT* expression level in *C. sinensis* L. was relatively high in winter and at high altitudes (Figure 4 and Figure 5), indicating that *CsHCT* has a high level of expression in low temperatures. The *CsHCT* expression level increased under low-temperature stress and decreased under high-temperature stress (Figure 6A). Thus, *CsHCT* expression is induced in low temperatures and may be involved in the defense pathways against low-temperature stress. Research demonstrated that HCT expression is regulated by biotic and abiotic stresses, thereby increasing stress tolerance in plants [6]. Our data indicated that *CsHCT* expression in *C. sinensis* L. can be induced with low-temperature, high-salinity, and drought stresses, and the expression level was particularly high with ABA treatment (Figure 6B).

Phytohormone ABA involved in stress tolerance in plants can be divided into ABA-dependent and ABA-independent signaling pathways. ABA-dependent pathways transmit signals through ABA, thereby activating downstream transcription factors such as the ABRE-binding factor/ABA-responsive element-binding protein, myelocytomatosis, and myeloblastosis to regulate plants’ stress tolerance [17,18]. This study demonstrated that *CsHCT* expression was induced by the abiotic stresses of low temperature, high salinity, and drought as well as ABA treatment. The signals of the three stresses may be transmitted through ABA-dependent pathways and may affect the expression of transcription factors of genes associated with the regulation of secondary metabolism. In this study, our results demonstrated the relationship between CsHCT expression and hormone signaling in oolong tea plants and may help improve the quality and possible health benefits of tea in the future.

## 5. Conclusions

Taken together, our study results indicated that *CsHCT* cloned from the Chin-Shin Oolong tea plant may be involved in tea plants’ response to abiotic stresses (i.e., low temperature, high salinity, and drought) and various hormone signaling pathways that affect tea plants’ secondary metabolic pathways.

## Abbreviations

ABA	Abscisic acid
ACC	1-aminocyclopropane-1-carboxylic acid
BAHD	BAHD actyltransferase
HCT	Hydroxycinnamoyl transferase
MeJA	Methyl jasmonate
qRT-PCR	quantitative real-time polymerase chain reaction
SA	Salicylic acid
